# A comparative investigation of variant calling and genotyping for a single non-Caucasian whole genome

**DOI:** 10.21203/rs.3.rs-2580940/v1

**Published:** 2023-03-06

**Authors:** HyeonSeul Park, JungSoo Gim

**Affiliations:** Chosun University; Chosun University

## Abstract

Most genome benchmark studies utilize hg38 as a reference genome (based on Caucasian and African samples) and ‘NA12878’ (a Caucasian sequencing read) for comparison. Here, we aimed to elucidate whether 1) ethnic match or mismatch between the reference genome and sequencing reads produces a distinct result; 2) there is an optimal work flow for single genome data. We assessed the performance of variant calling pipelines using hg38 and a Korean genome (reference genomes) and two whole-genome sequencing (WGS) reads from different ethnic origins: Caucasian (NA12878) and Korean. The pipelines used BWA-mem and Novoalign as mapping tools and GATK4, Strelka2, DeepVariant, and Samtools as variant callers. Using hg38 led to better performance (based on precision and recall), regardless of the ethnic origin of the WGS reads. Novoalign + GATK4 demonstrated best performance when using both WGS data. We assessed pipeline efficiency by removing the markduplicate process, and all pipelines, except Novoalign + DeepVariant, maintained their performance. Novoalign identified more variants overall and in *MHC* of chr6 when combined with GATK4. No evidence suggested improved variant calling performance from single WGS reads with a different ethnic reference, re-validating hg38 utility. We recommend using Novoalign + GATK4 without markduplication for single PCR-free WGS data.

## Introduction

The development of next-generation sequencing (NGS)^[1]^ has facilitated high speed and low cost analyses of personal genomes^[2–4]^. Increasing options for analyzing personal genomes and identifying variants are now available, along with additional analytical tools. Choosing the optimal analysis pipeline based on speed and accuracy can influence subsequent analyses^[5, 6]^. The next aim is to determine the appropriate pipeline for specific ethnicities, since many differences have been observed between different ethnic groups^[7]^.

To construct optimal variant calling pipelines, benchmark studies have been conducted using a combination of several analytical methods^[8–11]^. Most benchmark studies use Caucasian sample data, also known as NA12878, and hg38 or hg19 as reference genome, which is also Caucasian-centric. The hg38 or hg19 is an accurate, precise and extensive general human reference genome ^[12, 13]^, but the optimality of the variant calling pipelines from obtained from those studies is not free from criticism.

Although the reference genome is meant to be a standard, its practical implications are not clearly defined. For example, allelic diversity within the reference genome is not an average of the global population (or any population), but rather contains long stretches that are highly specific to one individual^[12]^. Sequencing a large number of human DNAs revealed the presence of numerous rare variants^[14, 15]^. In particular, when using these general reference genomes, variants that may be observed only in a particular population can be easily missed. Approximately 10% of DNA sequences in the pan-assembly of the African genome are missing in the GRCh38 reference; therefore, there is a need for ethnicity-specific references as well as multi-ethnic panels.

**Figure 1:**
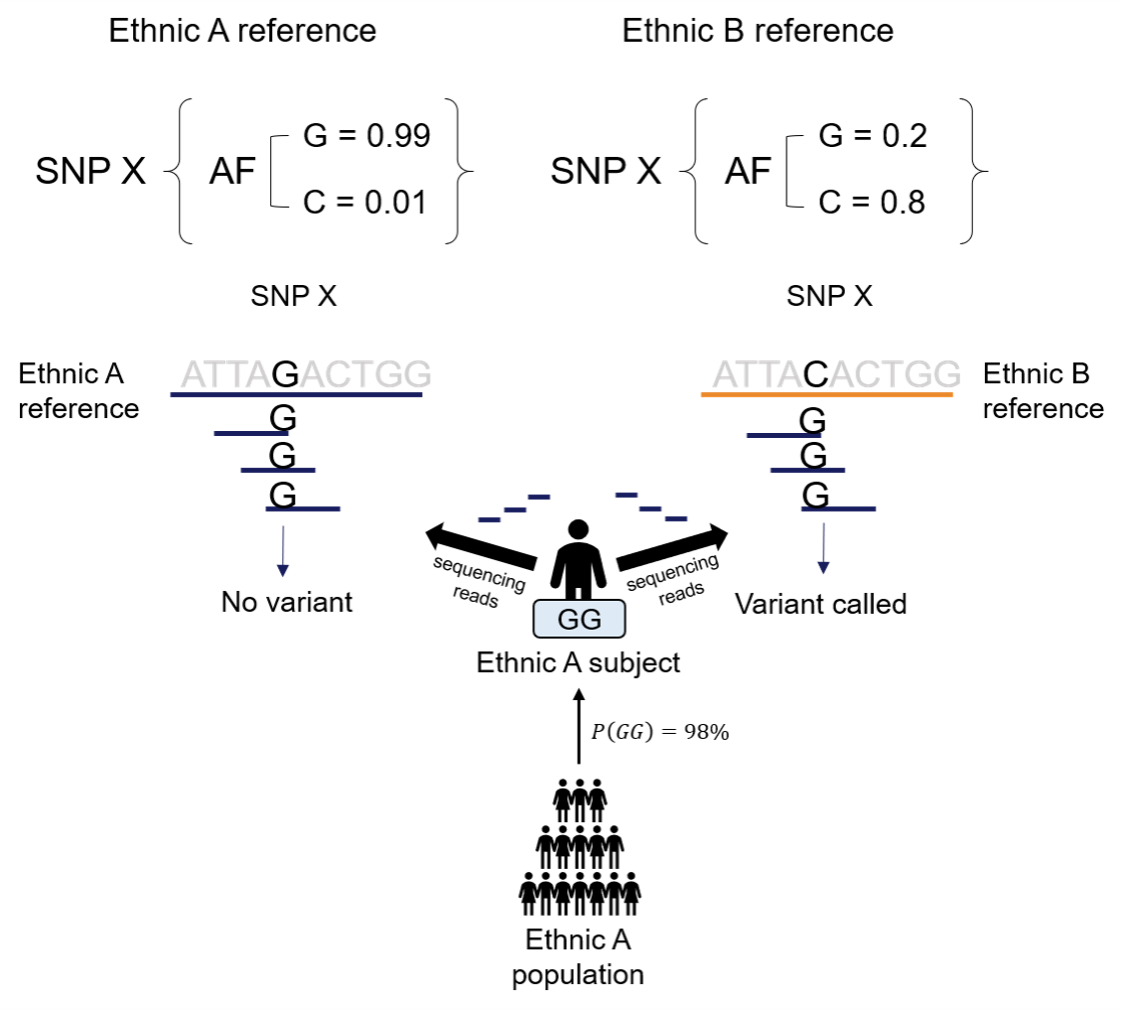
A schematic diagram showing the necessity of a multi-ethnic reference genome Variant calling when using a reference genome of an ethnic group with different allele frequencies for the same random SNP X. Example: The allele frequency of G and C for SNP X in ethnic group A is 0.99 and 0.01, respectively, and the most likely genotype is GG with a frequency of 0.98. Using WGS data from a subject in ethnic group A, Ethnicity A reference: 0.99 × 0.98 = 0.9702 Ethnicity B reference: 0.80 × 0.98 = 0.784

Multi-ethnic panels include methods for newly assembling a reference genome and comparatively analyzing ethnicity-specific variants at each position^[16–18]^. They can also be analyzed using an ethnicity-specific reference genome, although they cannot be considered as standards^[12]^. When using the reference genome of an ethnic group with different allele frequencies for the same random SNP X, the variants called may be different. When analyzing a WGS, if a variant is called using a reference genome corresponding to the same ethnicity, and if the sample size of the sequenced samples is not large, the variant will likely not appear at that position, whereas for other ethnic groups, it may. Furthermore, if the ethnicity of the reference genome and analyzed subject is different, the allele frequencies are different, and more variants are identified. Therefore, we suggest that general reference genomes should account for more ethnic variants (Fig. 1).

In this study, we investigate the effect of the ethnicity of the reference genome on variant calling in a heterogeneous sample. For that purpose, we compared eight different variant calling pipelines for WGS of a Korean sample (and NA12878 as well) with the true set of variants genotyped using Sanger sequencing and a microarray platform using two reference genomes from different origin: hg38 and Korean.

## Results

### Comparison of multiple pipelines for analyzing WGS data from different origin

**Figure 2:**
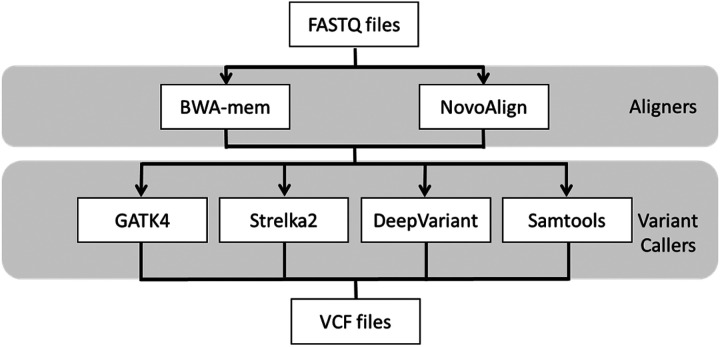
**Work flow used for comparative analyses** The work flow of combinations using different aligners and variant callers for germline variants. The following eight pipelines were constructed by combining aligners and variant callers: BWA-mem + GATK4, BWA-mem + Strelka2, BWA-mem + DeepVariant, BWA-mem + Samtools, Novoalign + GATK4, Novoalign + Strelka2, Novoalign + DeepVariant, and Novoalign + Samtools.

**Figure 3:**
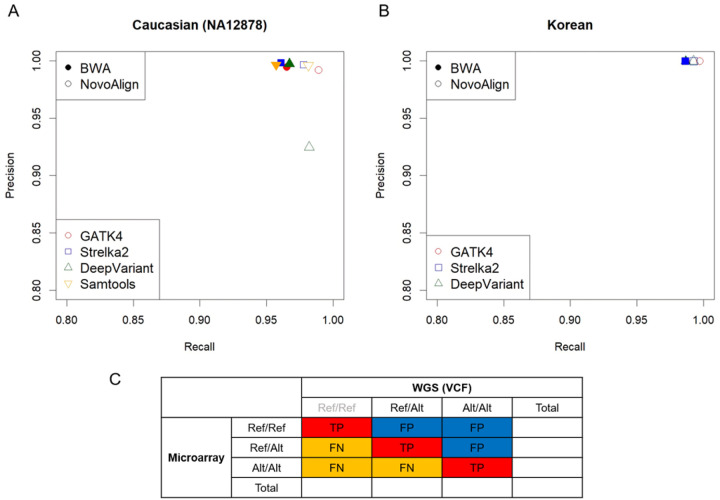
SNP performance using multiple pipelines Colors indicate the variant callers, and the difference between the color-filled and unfilled shapes is the use of the aligners. The x-axis indicates recall, while the y-axis indicates precision. **(A)** Performance of a SNP in multiple pipelines using Caucasian data (NA12878). **(B)** Performance of a SNP in multiple pipelines using Korean data. **(C)** Definition of performance based on genotype concordance using microarrays and WGS VCF files.

Eight pipelines were compared to determine their performance when using human WGS data (Fig. 2). When the performance of each pipeline was compared between the Caucasian data NA12878 and the data from Korean subject1, using the hg38 reference genome, there were no significant differences (Fig. 3).

The Caucasian data, NA12878, had corresponding VCF data provided by Illumina, which facilitated performance comparison. The recall value was the highest when Novoalign + GATK4 was used, and the precision value was highest with BWA-mem + Strelka2. The best performance, based on both precision and recall, was achieved with the Novoalign + GATK4 pipeline (Fig. 3).

For the Korean subject1 data, we analyzed using each pipeline excluding Samtools, which had the longest runtime among the eight pipelines used for comparing performance (Supplementary Figure S1). Unlike the NA12878 data, the Korean subject1 data did not have a gold standard reference; therefore, we used microarray data for the performance comparison. The results indicated no significant differences in pipeline performance but showed that precision was high for the BWA-mem + Strelka2 pipeline while recall was high for the Novoalign + GATK4 pipeline. The Novoalign + GATK4 pipeline showed the best performance when both precision and recall were considered (Fig. 3B). When Novoalign was used as an alignment tool, more variants were identified than those using BWA-mem; in particular, the Novoalign + GATK4 pipeline detected more variants compared to other pipelines. Similar results were obtained when data from the other two Korean subjects were used (Supplementary Table S1).

Among the variant call tools, the runtime for BWA-mem was shorter than that for Novoalign, and was fastest for Strelka2 (Supplementary Figure S1). BWA-mem took approximately 54 minutes to index the reference file and 1 hour 22 minutes for alignment. In contrast, Novoalign took 1 minute to index the reference file and approximately 5 hours for alignment. Since alignment, switching to the bam file, and the sorting process took approximately 20 minutes using Samtools, we set the thread to 64. To obtain a variant call using a BWA-mem mapped file, GATK4 took 5 hours, Strelka2 took 33 minutes, and DeepVariant took approximately 7 hours. When proceeding with a variant call using a file mapped in Novoalign, GATK4 took 4 hours, Strelka2 took 30 minutes, and DeepVariant took 7 hours and 30 minutes. In the case of Strelka2, the required time differed depending on the presence or absence of the BED file, but with the NA12878 data, if the BED file was not included, a variant call of the file mapped with BWA-mem took 40 minutes; in contrast, Novoalign took 5 hours 45 minutes (Supplementary Figure S2). Therefore, when using Strelka2, including a BED file can facilitate faster runtimes.

To verify the results of the pipeline performance analyses, Sanger sequencing was performed with the Korean subject1 data. The results showed that the concordance rate was higher when Novoalign was used as the alignment tool than the BWA-mem and was the highest when GATK4 was used as the variant caller (Table 1).

### Variant calling with an alternative Korean reference

The reference genome mostly built with a Caucasian. Thus, we proceeded with the analysis using a Korean reference genome as well as ‘the standard reference genome’, or hg38. We assessed the differences when the Korean genome was set as the reference in the Caucasian and Korean WGS analysis versus when the hg38 reference genome was used.

When using the Korean reference genome, the SNP precision and recall of the BWA-mem + Strelka2 pipeline with Caucasian WGS data were 0.936296 and 0.490368, respectively; the recall was very low, while in INDELs, the precision and recall were 0.38198 and 0.425591, respectively.

**Figure 4:**
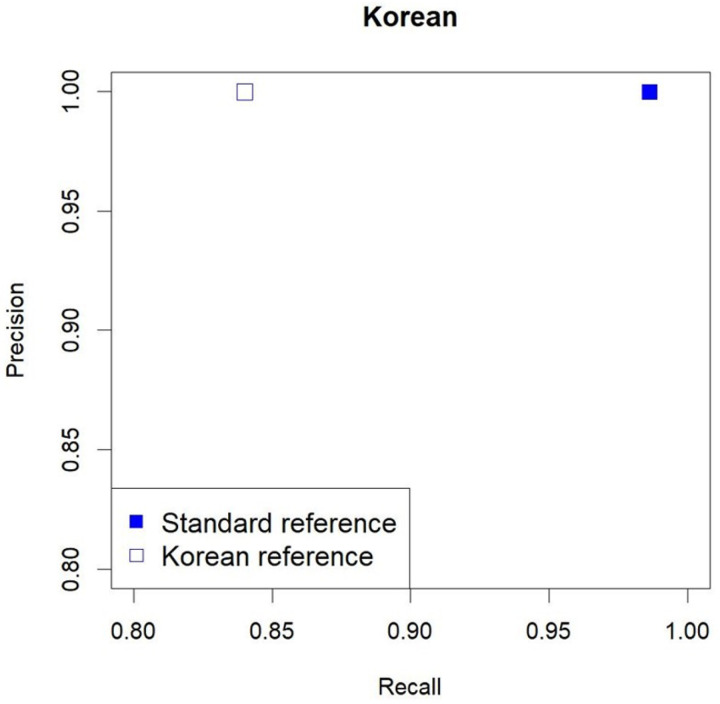
Performance of the BWA-mem + Strelka2 pipeline according to the reference genome The squares signify the BWA-mem + Strelka2 pipeline and show the performance of the Korean WGS data when analyzed using the general (standard) and Korean reference genomes. The x-axis indicates recall, while the y-axis indicates precision. Filled and unfilled squares indicate use of general reference genome and the Korean reference genome, respectively.

For the Korean WGS data, we compared the precision and recall with the microarray data. In a previous study using a general reference genome, among the 142,037 variants that matched the rsIDs of the WGS VCF file and microarray data, 141,885 variants showed exactly matched genotypes. When using the Korean reference genome, there were 77,679 variants with concordant rsIDs and 77,499 variants with concordant genotypes (Supplementary Table S2). In total, 440,188 SNPs were identified in the microarray data, with precision and recall values of 0.999691 and 0.986326, respectively, for the general reference and 0.999762 and 0.8399762, respectively, for the Korean reference (Fig. 4).

**Figure 5:**
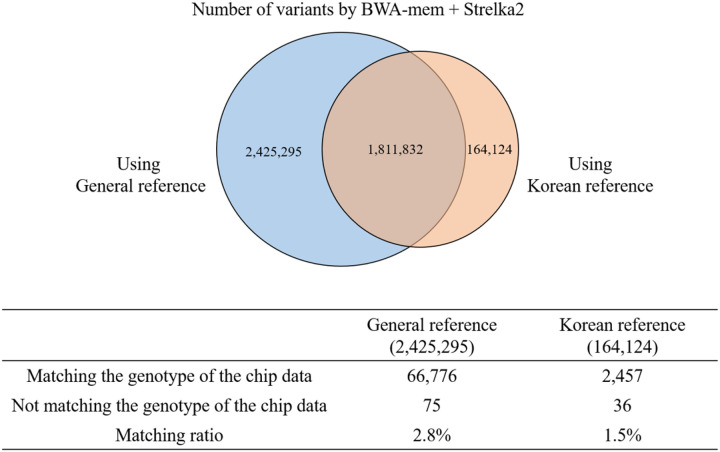
Number of variants obtained by BWA-mem + Strelka2 when using different reference genomes Using the general and Korean references, the BWA + Strelka2 pipeline called 4,237,127 and 1,975,956 variants, respectively. Of these, 1,811,832 variants overlapped; furthermore, there were 2,425,295 non-overlapping variants from the general reference and 164,124 non-overlapping variants from the Korean reference. The table shows the cases where the chip data and genotype matched among the variants that did not overlap. Of the 2,425,295 non-overlapping variants using the general reference, 66,776 variants matched (approximately 2.8%) with the chip data genotypes, and using the Korean reference, 2,457 genotype matches (approximately 1.5%) were found among the 164,124 variants.

We compared variants called using the same pipeline and different reference genomes and inferred that the general reference genome was better than the Korean reference genome in the context of performance. When using a general reference genome rather than a Korean reference genome, the pipeline could call more variants. Furthermore, 1,811,832 variants were found to overlap. When checking whether chip data and genotype showed concordance among non-overlapping variants, we found a 2.8% match rate when using the general reference (Fig. 5).

### Comparison with or without markduplicate

The markduplicate process is necessary to remove PCR duplicates that are introduced during NGS library construction. However, since PCR-free libraries are often used for subsequent sequencing, we assessed whether the markduplicate step is still necessary.

We compared the performance of Korean WGS data sequenced using the PCR-free library with and without the markduplicate step. In our computation environment, it respectively took 270 and 540 minutes (respectively corresponding to 44 ± 15% and 51 ± 10% of all anlysis time) running the markduplicate process on a BAM file mapped using BWA-mem and Novoalign when using Korean data. Therefore, this step increases the overall analysis time (Supplementary Figure S3).

In addition, the time was measured using NA12878 data to compare runtime by option. The runtimes when the Java option (-Xmx, -Xms) was given at 8g and 32g were compared. When given at 8g, the results were somewhat clearer^[28]^. It took 269 and 233 minutes to run markduplicate process with a BAM file created by Novoalign and BWA-mem, respectively. The 32g option took 283 and 288 minutes, respectively. These results demonstrated that the markduplicate process takes up a significant amount of time during preprocessing and identified that it is appropriate to use the 8g option when using the markduplicate process.

**Figure 6:**
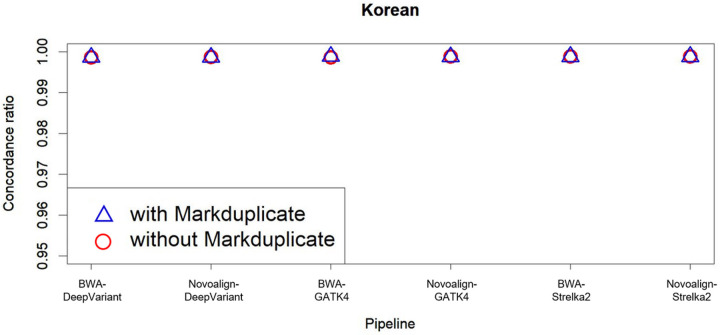
Concordance ratio according to markduplicate usage in multiple pipelines The x-axis shows the pipeline, while the y-axis shows the concordance ratio. The concordance ratio indicates the ratio between the matching variant rsIDs in the microarray and Korean WGS data with exact genotype concordance (concordance ratio = rsIDs with concordance in genotype/matching rsIDs among variants). The blue and red triangles indicates when the variant is called with or without markduplicate use, respectively.

We then compared the performance of the pipeline with or without the markduplicate step, using Korean WGS data sequenced using the PCR-free library (Fig. 6). Comparing the match rate of the genotypes from the microarray chip and WGS VCF data confirmed that there were no significant differences based on whether markduplicate process was performed. These results agreed with those obtained using NA12878 (Supplementary Figure S4).

### Best pipeline for MHC region analysis

**Figure 7:**
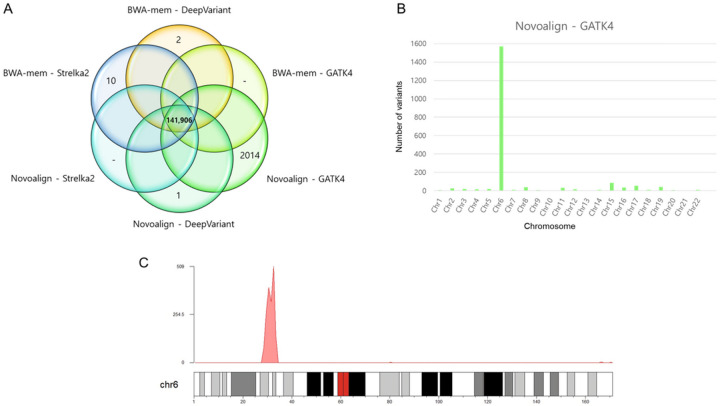
Variant distribution in only one pipeline **(A)** Each circle indicates the number of variants called in only one pipeline. **(B)** Distribution of variants in Novoalign + GATK4 **(C)** Distribution of the Novoalign + GATK4 variants at chromosome 6

We confirmed that using Novoalign as an alignment tool calls more variants than when BWA-mem is used. Based on the number of variants that appear in each pipeline alone, we found that when using standard references, the Novoalign + GATK4 pipeline calls far greater number of variants than other pipelines (Fig. 7A). When we identified the distribution of variants that appeared only in this pipeline, we confirmed that among the 2014 variants, 1568 variants originated from chromosome 6 (Fig. 7B). Among these, 1564 variants were found in the major histocompatibility complex (MHC) region (28,477,797–33,448,354, hg19) (Fig. 7C). This MHC region demonstrated a 99.87% match to the microarray chip and WGS genotypes (mismatches were detected only in two out of 1564 variants). The results were similar for the other two Korean subjects and for Korean subject1. Similarly, when the Novoalign + GATK4 pipeline was used, a large number of variants was detected (Supplementary Table S3); approximately 80% of the detected variants were in chromosome 6, and the majority belonged to the MHC region (Supplementary Figure S5). Additionally, the concordance ratio with the chip data was 99.83% and 99.94%, respectively, for Korean subject2 and subject3. Therefore, to analyze variants in the MHC region, we recommend using the Novoalign-GATK4 pipeline.

## Discussion

With the development of NGS technology, the cost of genome sequencing has decreased significantly, thus facilitating personal genome analysis and custom diagnostics. We hypothesized that it is insufficient to analyze genomes using the same pipeline without considering ethnicity, and therefore attempted to identify an optimal pipeline. In this study, we analyzed Caucasian NA12878 and Korean WGS data in their respective pipelines and compared their performances.

For NA12878, gold standard data was available to which WGS VCF files from the pipeline could be compared, whereas the Korean WGS data had no gold standard data. Therefore, the Korean WGS data were compared to microarray chip data to determine pipeline performance. However, since the Korean WGS data had microarray chip data which used Korean chips composed of Korean data-specific probes, many variants did not overlap with those in the WGS data. Therefore, when we compared the rsIDs concordant between the microarray chip and WGS VCF files for the Korean data, using Strelka2 resulted in a slightly higher rsID genotype concordance rate than using the other pipelines. However, the Novoalign + GATK4 pipeline showed the best performance when considering TP, FP, FN, precision, and recall. All pipelines showed good performance when comparing the match rates between variants. Novoalign-GATK4 showed the best performance for both Caucasian NA12878 and Korean subject1. This result is significant since the Novoalign-GATK4 pipeline, which did not perform well in several benchmark studies, had the best performance in this study.

We hypothesized that different ethnic references could improve the performance of variant calling from single WGS reads. For single Korean WGS data, we found that using reference genomes of other ethnicities without using the reference genome corresponding to the ethnicity of interest helps to identify more variants. When using the hg38 and Korean reference genomes, the precision was not significantly different, yet the recall values were 0.986326 and 0.8399762, respectively, indicating that the reference genome was different. Therefore, using hg38 reference genome improved performance; however, the Korean reference genome used for comparison was not sufficient; thus, further studies on comparing reference genomes are warranted. This reference genome, created in 2015, has not been updated.

In this study, we observed several novel findings. First, during WGS data analysis, we assessed pipeline reconstruction by comparing the performance with and without the markduplicate process. We used data sequenced with PCR-free libraries; therefore, we expected no significant difference in performance even if the markduplicate process was excluded. The performance did not differ significantly with and without the markduplicate process; rather, the results of experiments with the NA12878 data showed that the Novoalign + DeepVariant pipeline performed better without markduplicate than with (Supplementary Table S1). Recently, a PCR-free library was used for NGS analysis; hence, these results will facilitate more effective analyses in the future.

Second, the MHC region on the short arm of chromosome 6 is highly polymorphic, gene-dense, and associated with more diseases than any other genome region. Furthermore, it encodes human leukocyte antigen (HLA) proteins and is one of the key immunogenic regions. Recent algorithmic and technological advances have facilitated the identification of genetic variations in the MHC region. In this study, we found a specific pipeline in which several variants corresponding to the MHC region can be identified^[29, 30]^. When comparing the alignment tools Novoalign and BWA-mem, a high number of variants was called when mapped using Novoalign in the overall pipeline comparison.

The pipeline with no overlapped variants among the six pipelines was also identified. Of the six pipelines, the Novoalign + GATK4 pipeline called 100-fold more variants than the remaining pipelines and that these variants were primarily in the MHC region. Therefore, when studying MHC regions or HLA genes, we recommend using the Novoalign + GATK4 pipeline. In summary, we recommend using the Novoalign + GATK4 pipeline for SNP calling and excluding the markduplicate step when sequencing is performed via a PCR-free library. As we did not examine INDEL separately using Korean data, this pipeline may have limitations in analyzing INDELs; however, our results identified the best pipeline for SNP analysis. This study has some limitations. We used only one Korean subject and conducted limited Sanger sequencing for variants in the one subject due to limited financial resources. Further studies with additional subjects and more comprehensive sequencing are warranted

## Materials And Methods

### WGS data

We used Caucasian and Korean data to compare pipeline performance with respect to ethnic differences. The Caucasian sequence data was retrieved from the NA12878 FASTQ file from the SRA database SRR8454589. This file had approximately 30X depth and was obtained using an Illumina Novaseq6000^[8]^. For the Korean data, sequencing was performed at 30X depth using whole blood from a healthy subject. Briefly, the NGS library used was the Truseq PCR-Free Prep library kit, a PCR-free library^[19]^, and it was sequenced in an Illumina Novaseq6000 in a paired-end 150-bp format.

### Reference genome for WGS analysis

Using WGS data for analysis, we used the reference genome (Homo_sapiens_assembly38.fasta) provided by GATK and the Korean reference genome created in 2015^[13]^.

### Pipeline for WGS data analysis

The pipeline used was recombined with the tools used for the best performing pipeline from the benchmark studies for NA12878. The following combinations were used: BWA-mem and Samtools^[9]^, BWA-mem and DeepVariant, GATK4^[11, 20]^, BWA-mem and Strelka2^[8]^, BWA-mem and DeepVariant, NovoAlign and DeepVariant, BWA-mem and Samtools, and NovoAlign and Samtools^[10]^. Among these combinations, BWA-mem (version 0.7.17)^[21]^ and Novoalign were used for alignment, and GATK4 (version 4.1.8), Strelka2 (version 2.9.10)^[22]^, DeepVariant^[23]^ and Samtools were used for variant calling. Finally, eight pipelines were constructed using these combinations. GATK used the Baserecalibrator, ApplyBQSR, Haplotypecaller, FilterVCF, MergeVCF, and GenotypeGVCFs options. Strelka2 used the option to use the BED file along with the basic options and the speeding up option. DeepVariant used the remaining threads as a default. Samtools used GATK3’s RealignerTargetCreator, IndelRealigner, BaseRecalibrator, PrintReads, and then mpileup^[24]^. We initially tried to use Samtools mpileup; however, we ended up using bcftools mpileup as recommended by the manual (http://www.htslib.org/doc/Samtools-mpileup.html).

### Definitions for performance comparison of pipelines for WGS data

The eight pipelines were used to analyze Caucasian and Korean WGS data, and variant call format (VCF) files were used to compare the pipeline performance. Several studies have obtained NA12878.VCF files from Illumina FTP for the Caucasian data (NA12878). For performance comparison, we used the hap.py tool to calculate the performance indicators, including^[8, 9]^:

Precision:TP/(TP+FP)

Recall:TP/(TP+FN)

We defined true positive, true negative, false positive, and false negative as follows:

true positive (TP): variants called by a pipeline being the same genotype as the gold standard data;

true negative (TN): reference alleles in high confidence regions other than the gold standard variant set;

false positive (FP): variant called by a pipeline but not of the same genotype as the gold standard data;

false negative (FN): gold standard variants with high confidence that were not called by a pipeline.

The Korean WGS data were analyzed using six pipelines, excluding Samtools from the Caucasian data analysis (due to long computing duration). Owing to the absence of a Korean data gold standard, performance was compared using microarray-based genotypes of the same subjects. The resulting variants of each pipeline were annotated with a SNP ID (rsID) using bcftools annotate, then SNPs that overlapped between the VCF and microarray-based genotypes were selected, and genotype combinations from each were combined into a 3 × 3 contingency table for performance calculation^[25]^.

The Ref/Alt concordance called in two different platforms (microarray and WGS) was compared. Variants not called in WGS but genotyped as Ref/Ref in the microarray were manually called Ref/Ref with the major allele of the reference sequence. Variants that were only detected in the WGS and microarray were regarded as FP and FN, respectively. Variants were classified as TP when two platforms called concordant matches, as described by Kishikawa et al.^[25]^.

### Comparison of concordance with and without PCR duplicate removal

The pipelines used for the analysis underwent a Samtools view step that replaced the SAM file with a BAM file after alignment, a Samtools sort step that sorted the created BAM file, and an index step. A markduplicate process was then performed using the Picard tool [https://broadinstitute.github.io/picard/] to remove the PCR duplicates amplified during the NGS library preparation process.

Most studies have been using PCR-free libraries for sequencing, but variant call processes include a markduplicate step that removes PCR duplicates^[26]^. In this study, we compared the performance of the pipelines with and without the markduplicate step. Precision and recall values with or without the markduplicate step were compared for the Caucasian data. For the Korean data, concordance among genotypes with the microarray data was compared for the VCF files. For the variant concordance rate, the percentage of variants with matching rsIDs, which completely matched the genotypes, was calculated.

### List of Sanger sequencing variants used for validation

Sanger sequencing was performed to validate the results^[27]^. The variant set used in Sanger sequencing was chosen based on the following criteria:

- Variants from only one variant caller when using the same alignment tool
- Variants from the same variant caller when using the different alignment tools
- Variants detected in only one pipeline

### Regional annotation for SNPs enriched in chromosome 6

The annotated VCF files obtained from the results of all the pipelines were compared with the rsIDs of the microarray data. Only variants with matching rsIDs for the VCF files and microarray data were selected. Variants overlapping with other pipelines and those without overlap were separated, and variants that did not overlap and appeared specifically in only one pipeline were confirmed. We then identified the characteristics of the corresponding variants.

## Figures and Tables

**Table 1:** Sanger sequencing data of Korean subject1

Pipeline		Number of variants	Genotype concordance	Concordance ratio
Alignment	Variant Caller			
BWA-mem	DeepVariant	14	10	0.7143
	GATK4	11	9	0.8182
	Strelka2	15	12	0.8
NovoAlign	DeepVariant	19	15	0.7895
	GATK4	37	32	0.8649
	Strelka2	21	18	0.8571

## Data Availability

FASTQ file and the genotype list from Sanger validation are publicly available (SRR22944222). Details can be found at https://dataview.ncbi.nlm.nih.gov/object/PRJNA917194?reviewer=s6fvbckl66d5p15rfohl60p9sm
